# “You can kind of just feel the power behind what someone's saying”: a participatory-realist evaluation of peer support for young people coping with complex mental health and substance use challenges

**DOI:** 10.1186/s12913-022-08743-3

**Published:** 2022-11-16

**Authors:** Tanya Halsall, Mardi Daley, Lisa Hawke, Joanna Henderson, Kimberly Matheson

**Affiliations:** 1 University of Ottawa Institute of Mental Health Research at The Royal, 1145 Carling Avenue, Ottawa, Ontario K1Z 7K4 Canada; 2 Department of Neuroscience, Carleton University, 1125 Colonel By Drive, Ottawa, ON K1S 5B6 Canada; 3 LOFT Community Services, 721 Bloor St. W Suite 103, Toronto, ON M6G 1L5 Canada; 4 Centre for Addiction and Mental Health, 80 Workman Way, Toronto, ON M6J 1H4 Canada; 5 Department of Psychiatry, University of Toronto, 250 College Street, Toronto, ON M5T 1R8 Canada

**Keywords:** Community based research, Mixed methods, PAR—participatory action research, Qualitative evaluation

## Abstract

**Background:**

Youth peer support, as a practice that aligns youth engagement and participatory approaches, has become increasingly popular in the context of youth mental health services. However, there is a need for more evidence that describes how and why youth peer support practice might be effective. This study was designed to examine a peer support service for youth experiencing complex challenges with mental health, physical health and/or substance use to better understand key features and underlying mechanisms that lead to improved client outcomes.

**Methods:**

We applied a hybrid realist-participatory approach to explore key issues and underlying theoretical assumptions within a youth peer support approach for young people (age 14–26) experiencing complex mental health and substance use challenges. We used semi-structured interviews and focus groups with staff, including peers (*N* = 8), clinical service providers and administrative staff (*N* = 15), to develop the theories and a client survey to validate them. Our qualitative thematic analysis applied a retroductive approach that involved both inductive and deductive processes. For the client survey (*N* = 77), we calculated descriptive statistics to examine participant profiles and usage patterns. Pearson correlations were examined to determine relationships among concepts outlined in the program theories, including context, mechanism and outcome variables.

**Results:**

Our analyses resulted in one over-arching context, one over-arching outcome and four program theories. Program theories were focused on mechanisms related to 1) positive identity development through identification with peers, 2) enhanced social connections, 3) observational learning and 4) enhanced autonomy and empowerment.

**Conclusions:**

This study serves as a unique example of a participatory-realist hybrid approach. Findings highlight possible key components of youth peer practice and shed light on the functional mechanisms that underlie successful peer practice. These key components can be examined in other settings to develop more comprehensive theories of change with respect to youth peer support and can eventually be used to develop guidelines and standards to strengthen practice. This research contributes to an expanding body of literature on youth peer support in mental health and connects peer practice with several social theories. This research begins to lay a foundation for enhanced youth peer support program design and improved outcomes for young people experiencing complex mental health and substance use challenges.

## Background

There is an increasing interest and uptake of youth peer support services within youth mental health [1, 2], particularly as an approach that is embedded within a range of integrated services (see [3–6]). Peer support has been defined as a “supportive relationship between people who have a lived experience in common” ([7], p. 7). Since peer support engages individuals who share lived experience with clients, it imparts the benefits of increased critical insight from this common background, which aligns with patient-oriented and participatory principles [3, 8].

Although there is an expanding body of literature that describes the benefits of adult peer support programs, including several reviews [9–11], there is limited research on youth peer support interventions [1, 12, 13]. Specifically, there continues to be a need to identify which characteristics of peers are most influential in supporting client improvements [14], including the contexts that enhance peer support effectiveness [10, 15], the unique contributions peer services offer as a complement to mental health services [1, 9, 10], and for whom are these services beneficial [15]. Further, there is a need to better understand the mechanisms of influence that support positive outcomes within youth peer support [1, 15, 16].

This paper describes a hybrid realist-participatory evaluation approach that was designed to address the above research gaps. This study examines a peer support program for youth with complex mental health and social challenges to identify “what works, for whom, how, why and in what circumstances?” [17, 18].

### Peer support in youth mental health

Peer support in youth mental health represents a specific strategy that aligns with youth engagement and other integrative approaches that utilize youth voice to inform and improve services for young people [8, 19]. In the field of mental health, youth engagement relates to ‘empowering all young people as valuable partners in addressing and making decisions that affect them personally or that they believe to be important’ ([20] , p. 5). Youth peer support leverages the insights gained through lived experience of mental health and related challenges and cultivates the integration of this awareness with in-depth knowledge of practice and service provision to offer strengthened clinical insight and the potential to influence client recovery [3]. Youth peer support is often positioned as a complementary service within more formalized supports [6, 21] and peer roles often involve a range of skills and responsibilities, including skill-building, emotional support, service navigation, mental health education and promotion, action planning, engagement, coordination support and evaluation [1].

### Theoretical underpinnings of youth peer support

There are several studies that have explored theoretical explanations for how youth peer support programs might be effective. For example, research has identified a range of positive impacts from receiving youth peer support services, including increased coping, social connection, hope, empowerment and recovery [22–25], all of which are implicated within Social Cognitive Theory [26, 27]. This theory has been used to explain how the learning process occurs and suggests that new skills and knowledge are often acquired through social observation [27]. It is through these social learning mechanisms that peers may offer increased benefits to clients through sharing coping strategies [12] and through role modelling effective functioning [28]. Learning might be reinforced further through the observation of consequences related to peer behaviours [28]. As such, clients can vicariously learn from peers as role models who have advanced in their recovery and experienced positive health outcomes.

Self-efficacy is an important concept within Social Cognitive Theory. It has been defined as an individual’s judgement of their own personal ability to succeed [26]. This concept has often been applied to peer support, particularly with respect to coping [11, 14, 29–31]. Through peer support, clients may develop stronger self-efficacy by following peer guidance and experiencing success in their own recovery and coping skills [3].

Another theory that may be relevant for explaining how peer support services are effective is Social Identity Theory [32]. This theory suggests that group membership and the affective meaning associated with that group serve to define an individual’s own self-concept. This process has implications for the self-stigma associated with mental illness as well as how peer support might help to overcome this stigma. Group-focused interventions designed to decrease self-stigma have been found to be effective for individuals coping with mental health challenges [33]. Further, interpersonal connection with individuals coping with mental health challenges has been associated with improved attitudes with respect to stigma [34] and identifying with groups affected by mental health challenges may reinforce self-esteem when encountering a stigmatizing situation [35]. Peer support has been associated with both reduced client self-stigma as well as reduced stress related to stigma [24]. It is important to develop a better understanding of the processes that diminish the impacts of stigma as it has been identified as one of the main barriers to the delivery of mental health care [36]. Stigma has been recognized as a significant concern for young people and as a deterrent in seeking help for mental health issues [37–39].

A third theory that has been discussed as having relevance for peer support practice is Self-determination Theory [40, 41]. Basic psychological needs theory, one of the main mini-theories under the umbrella of the Self-determination Theory, suggests that there are three basic needs that must be satisfied in order to support overall wellbeing: 1) autonomy, 2) competence, and 3) relatedness [42]. In particular, autonomy and relatedness have been implicated as peer support practice often places a focus on supporting client independent identification of goals [40] and enhanced connections with others [41].

The Bioecological Model [43–45] can also be used to highlight key factors that might be associated with peer support practice to help explain how it functions. The bioecological model describes the role of interactions between context and individual that drive development. The bioecological model is relevant to peer practice as it highlights the significant strength of influence from developmental contexts, including the peer social context and the value of positive social connections [46].

### Purpose

This study was designed to examine a peer support service for youth experiencing complex challenges with mental health, physical health and/or substance use to better understand how and why it might be effective. We applied a hybrid realist-participatory approach to explore key issues and underlying theoretical assumptions within the Transitional Age Youth (TAY) peer support program. This paper focuses on the identification of program theories related to client outcomes. We used semi-structured interviews with staff to develop the theories and a client survey to triangulate the interview findings. The social cognitive theory and the social identity theory were applied to develop program theories and deductively tested through the analysis. The self-determination theory and the bioecological model were implicated through inductive analyses.

## Methods

### Realist-participatory approach

Realist evaluation is a theory-driven approach that typically uses mixed methods to capture the complex interactions within programs to identify how and why a program is effective [17, 18]. Within this approach, qualitative data collection is used to explore program processes and underlying theory, while quantitative data are used to examine outcome patterns [17]. Part of the realist evaluation approach involves the development of Context-Mechanism-Outcome-Configurations (CMOC). These are hypotheses that are designed to test theoretical assumptions regarding how a program works, and these assumptions take both contextual requirements and underlying mechanisms into account.

In contrast, youth participatory evaluation is an approach that engages young people in key decision-making within the evaluation process [47]. Involving youth peer supporters in research co-design strengthens impact through the inclusion of the lived experience of the client and service provider perspectives [3]. One peer from the LOFT Transitional Aged Youth department (TAY; MD) was a co-researcher and was involved in all key aspects of the study, including co-design of methods, facilitating engagement and coordination of staff, data collection, co-facilitation of presentations, analyses, interpretation of the data and authorship of publications. Other TAY staff, both peers and non-peers, were involved in key decisions and in reflecting on the findings (see [3]). Realist and participatory evaluation were combined in this study through an iterative approach that involved theory testing and adaptation to respond to questions of interest put forward by peers and other staff.

### Context

This research is the result of a partnership with the TAY department at LOFT Community Services in Toronto, Canada. The TAY program implements a range of programs and services, including case management, mental health supports navigation, social support, group drop-ins, campus-based services, supportive transitional housing and peer support for young people aged 14–26 years. Many of the young people who are served by the TAY program are experiencing challenges related to mental health, substance use, chronic physical illness or a dual diagnosis (developmental disability combined with mental health challenges) and a proportion of them are at risk of homelessness. The program was designed to enhance client life skills to support autonomy and achievement of personal goals and wellness. This study protocol has been approved by the Royal Ottawa Health Care Group Research Ethics Board (REB# 2019007). Informed consent was received from all participants and all procedures were performed in accordance with relevant guidelines.

### Qualitative procedures

The first round of semi-structured focus groups and interviews were facilitated with TAY program staff, including peers (*N* = 8), clinical service providers and administrative staff (*N* = 15). The focus group was completed at the end of an initial participatory workshop that was held during an all-staff meeting. During the workshop, the lead researcher presented the study background and general evaluation principles and led an exploratory discussion to gain insight regarding research questions of interest related to peer support. The participatory component of the research was developed based on the focus of this discussion and is described in Halsall [3]. Program staff (*N* = 23) attended the focus group and it lasted just over 45 minutes. Follow-up interviews were conducted to capture a greater depth of insight from key administrative staff (*N* = 1) and peers (*N* = 3), ranging from 50 to 90 minutes and conducted both in-person and over the phone. Realist interview guide questions were theory-driven and designed to distinguish context, mechanism and outcome patterns (e.g., Please describe how peer support services work? What kinds of characteristics are important to be a successful peer support worker? What can clients learn from youth peer support workers?).

An initial logic model was developed based on existing program documents, relevant literature and discussions with peer staff and this was used to develop three initial program theories or CMOCs (see [3]). The interviews and focus group were recorded and transcribed. Our analyses applied a retroductive approach that involved both inductive and deductive processes (see [48, 49]). An initial codebook was developed using constructs from Social Cognitive Theory [26] (e.g. self-efficacy, vicarious learning) and Social Identity Theory [32] (e.g. perceptions related to identity and social reference groups). Other codes were created using key concepts from the literature on peer support, including recovery, peer-client similarity, hope and stigma. The data were analyzed using QSR NVivo and an exploratory thematic analysis was used to generate new codes, as well as to deductively categorize codes within existing theoretical categories [50].

**Fig. 1:**
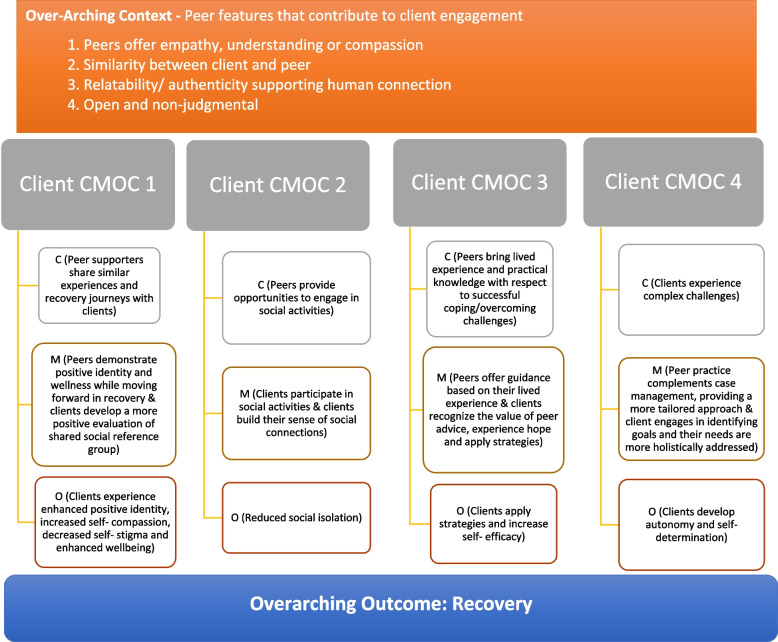
Diagram presenting over-arching context, over-arching outcome and the four program theories that were derived from the retroductive qualitative analysis

Interviews were divided among three coders who completed the initial coding separately. The three coders met and discussed codes identified and refined categories and definitions. Halsall completed a second round of coding to identify constructs that could be categorized under higher order codes of contexts, mechanisms and outcomes. A third round of coding was completed by Daley (peer co-researcher) to continue to refine categories and definitions. The three coders met to review final themes and to come to agreement on the initial CMOCs (see [3]) listed in Table 1. Only three CMOCs were identified in the initial round of analysis. These were later revised and a fourth program theory was elaborated. These are described in Fig. 1 (see Results section).

**Table 1:** Initial Context-Mechanism-Outcome-Configurations (CMOC)s that were developed through the first round of interviews and focus group

CMOC 1	**C** (Peer supporters share similar experiences and recovery journeys with clients) + **M** (Peers demonstrate positive identity and wellness while moving forward in recovery & clients develop a more positive evaluation of shared social reference group) ➔ **O** (Clients experience enhanced positive identity, decreased self-stigma and enhanced wellbeing)
CMOC 2	**C** (Peer supporters organize social events) + **M** (Opportunities to participate in social activities & clients build their sense of social connections) ➔ **O** (Reduced social isolation)
CMOC 3	**C** (Peers bring lived experience and practical knowledge with respect to successful coping / overcoming challenges) + **M** (Peers offer guidance based on their lived experience in addition to other mental health supports clients are receiving & clients recognize the value of peer advice and apply strategies) ➔ **O** (Clients’ experience success in applying strategies and increased self-efficacy / self-determination)

CMOCs are hypotheses designed to test theoretical assumptions regarding how a program works. “Context” is used to represent the necessary conditions that are implicated in supporting the functioning of the program. “Mechanism” relates to the underlying processes that function to generate program outcomes. Often, these involve participant interpretation and response to program offerings. “Outcome” represents the program impacts that result from the proposed context-mechanism interaction

These CMOCs were used to develop interview questions for a second round of interviews with peer staff only (*N* = 9; ranging from 40 to 65 minutes) that were conducted online by TH, MD and a third research assistant. The second round of interviews occurred about one year later. Therefore, some peers who had participated in the first round of interviews had left and several new staff were included. Revised interview guide questions were used to refine and test initial CMOCs (e.g., Have your previous experiences been helpful to you in supporting clients? If so, how? How do clients use this information? Has this resulted in any impact on clients…? What are the ways, if any, that peer support can enhance client social connections?). Halsall completed the first round of coding on the new data to integrate and build on new codes as well as to refine and expand existing CMOCs. Daley completed a second coding of the new data set and reviewed and refined codes and CMOCs. Halsall and Daley met to discuss and come to consensus on final themes, definitions and CMOCs. As with Dalkin and colleagues [48], language was refined to more clearly express key definitions and terminology used in peer support practice. For example, the “vicarious experience” code was revised to “skills transference”. This code was used to describe the knowledge and skills acquired by the client through peers sharing successful strategies they have used and insight gained through experience.

In order to validate the program theories, findings and CMOCs were presented to the TAY staff team to capture their feedback (*N* = 18) through a third participatory workshop that took place online. Program theories along with supporting findings were shared in a brief presentation and Mentimeter (Mentimeter.com) was used to capture audience agreement with program theories (see Table 2 for results).

**Table 2:** Validation results from staff feedback on four program theories

	*Program theory* n (%)			
	*CMOC1*	*CMOC2*	*CMOC3*	*CMOC4*
Level of agreement				
Agreed/strongly agreed	16 (89)	13 (76)	11 (65)	10 (62)
Neutral	1 (5)	1 (6)	5 (29)	6 (38)
Disagreed	0 (0)	1 (6)	0 (0)	0 (0)
Strongly disagreed	1 (5)	2 (12)	1 (6)	0 (0)

### Quantitative procedures

An online survey was administered to TAY clients to triangulate qualitative data regarding program theories and to explore outcome patterns. Participants were all current clients of the TAY program at LOFT and between the ages of 15–29 (*N* = 77). TAY staff invited clients to participate through peer groups and other services. Respondents were permitted to skip questions within the survey if they preferred not to respond. Participants received a $20 digital gift card as compensation for their time.

The Qualtrics software was used to administer the survey online and the survey was shared to clients through a link. Questions captured client demographics, process issues related with peer support services, perceptions about peer support services and positive mental health outcome indicators. We included questions to assess client perceptions with respect to context and mechanisms derived from the qualitative analysis with peers. Specifically, we asked “how well do you relate to your peer supporter?” (as an index of over-arching context). “How comfortable are you in engaging with your peer supporter?” (as an index of the over-arching context). “How much do you agree with the following statement: ‘I see my peer supporter as a positive role model’?” (as an index of the mechanism for CMOC1 and CMOC3). Response options to the first two questions were on a 4-point scale with a “don’t know” option (A lot (4), Somewhat (3), A little (2), Not at all (1)); The question that asked whether clients perceived their peer as a positive role model used a 5-point scale related to level of agreement (Strongly agree (5), Agree (4), Neither agree or disagree (3), Disagree (2), Strongly disagree (1)). Finally, we asked one question to measure client perception of CMOC2: “Has your participation in the LOFT peer support services helped you to make new social connections?” and if they responded yes, we asked them to please describe.

Single-item measures of self-rated mental health (ranging from poor (1) to excellent (5)) and life satisfaction (from very dissatisfied (1) to very satisfied (5)) were included to examine client wellbeing as an outcome [51]. In addition, five items were taken from the Youth Efficacy/Empowerment in Mental Health Scale–Self subscale (YES-MH [52];) and responses (using a 4-point rating scale; ranging from never (1) to always (4)) were averaged (Cronbach’s ⍺ = .82) to reflect self-efficacy as an outcome. A modified version of the Therapeutic Alliance Scale for Children: Youth version (TASC [53, 54];) measured the peer-client relationship and was used as an index of the relational context (12 items; Cronbach’s ⍺ = .89). This scale was modified so that respondents referred to their peer supporter rather than a therapist and included four response options ranging from “not like me” (1) to “very much like me” (4). To replace missing responses values were imputed from the mean of non-missing responses.

## Results

Our qualitative analysis resulted in four program theories that are outlined in Fig. 1. In contrast with typical program theories, we identified one over-arching context (peer features that contribute to client engagement) and one over-arching outcome (recovery) that appeared to be universally related to each CMOC.

### Over-arching context: peer features that contribute to client engagement

A fundamental part of the peer role involves creating an environment where clients felt accepted and comfortable sharing thoughts, feelings and experiences. This context set the tone for all functional interactions that could lead to client recovery. An important feature of building that context is peers sharing their lived experience with clients to demonstrate a shared understanding of a relevant challenge. Peers are set apart because of having a deep empathy for client experiences and this helps clients to feel more comfortable and willing to engage: “We’re not so different, so it becomes a safer space for you to have those interactions” (P1). “It’s such a unique way of connecting with people that no other role can connect on that level.” (P2).

Participants identified that with other clinicians, clients can feel like they are under a microscope and that they are being judged or evaluated. They may feel a greater distance and power differential in clinical interactions. In contrast, when clients are working with a peer, they feel a sense of acceptance and safety. In part, peer interactions were easier because clients had a sense that peers were coming from the same perspective and they felt more comfortable to open up. Part of the functionality of peer relationships is created by the fact that there is a shared similarity and this supports a unique contribution to recovery that other professional perspectives cannot offer: “Just by the fact that I do have this background in peer work, it is kind of like, we’re on the same level here, feel free to be yourself and to have problems.” (P3).

In the context of peer practice, peers and non-peers work together on the same teams to support clients. Often non-peers are positioned in more clinical evaluative roles, therefore, it is key for peers to set a more youth-friendly tone. “There’s usually a lot of relief in the room, where [clients] feel like they can maybe let down an extra few layers to a peer support worker.” (P5)

A lot of our clients actually have a lot of lived experience accessing service… so this might be their first time experiencing someone who doesn’t really come with a lot of that jargon or that really arms-length approach. And that could extend the feeling of being a little bit more accepted and safe, which makes them more likely to connect to our services and more likely to connect to others. (P6)

Part of creating the safer space for clients involves connecting through shared experiences, such as trauma, stigma, discrimination, homelessness and challenges related to mental health. Having these shared experiences increases the value of the peer relationship and the relevance and practicality of the advice they can offer. The TAY program serves a wide variety of clients coping with issues that range from human trafficking to eating disorders and clients may feel isolated as a result of these challenges.

If I'm working with someone who's been trafficked like, it's not something you want to talk about, per se, right? But being like, ‘Hey, you know what, I'm a survivor. Like, I get it’. And not that I get what you went through, but you don't have to explain it to me. I know why you wouldn't want to get out of bed in the morning. (P2)

Having a peer to facilitate communication and help them process these experiences can increase the likelihood for meaningful client engagement.

I remember times where I was speaking with somebody, and they had mentioned, like, disordered eating things and not like, it was something that they felt so uncomfortable talking about. But like, they said, knowing that, I had some experience with this as well, they felt less judgment for being able to talk about it. (P7)

### CMOC 1

The first program theory was developed based on Social Identity Theory [32]. It describes the process whereby clients recognize themselves in their peers and they also recognize that peers hold themselves in high regard, despite *and sometimes because of* experiencing similar challenges and issues (see Halsall T, Daley M, Henderson J, Hawke L, Matheson K.: “You can create a little bit more closure in your own story when someone really connects with it”: Exploring how involvement in youth peer support work can promote peer development, submitted). This understanding helps clients to develop a more positive sense of identity through their belonging to a shared reference group that may be characterized by a trait they typically feel stigmatized for. Participants talked about the self-acceptance and positive regard that peers hold and how this supports clients in developing a more positive self-regard: “It really rings true, it doesn’t feel like something that is just coming from like a textbook. It’s something that someone’s telling you about, like their journey, and … it’s easier to be more gentle on yourself.” (P8) “I’m not just saying I understand for the sake of saying I understand, you know? I think that really inspires them to be more than their diagnosis, to redefine their identity, again, to refine themselves in their recovery.” (P5)

I think it's just like having someone that you work with and you have respect for and seeing, and understanding that they have gone through some similar hardship as you um and they are doing okay. That's like really, I think affirming, for a lot of people. (P9)

The ability for the stigma within to kind of, like unfold itself and to feel like you're kind of out of this box that society puts us in. Or even oneself can, with shame, can just reveal this really like empowered feeling towards oneself. (P8)

Non-peer staff also recognized that they could perceive this process unfolding within peer practice.

I do also see a lot of [peers] just fully embrace some of their challenges, and so showing up so honestly about themselves, I think that sends some really accepting messages out… and clients feel that and they feel more accepted. (P6)

### CMOC 2

The second program theory is focused on the potential impacts that peer support can have on clients’ sense of social inclusion. Although peer interactions with clients are not friendships, they contained elements that more closely emulated interactions that would be expected in normal social relationships. Skills that were developed included practicing social awareness, communication skills and getting more comfortable in social situations.

Being able to like talk about certain things because you feel like someone can relate to you, gives you some sense of how to navigate certain friendships and relationships and what not. (P9)

Part of the peer role at TAY is to create opportunities for social interaction, such as during drop-ins, within group settings and out in the community.

I think that as a peer I was able to... show people some ways of how to interact … I really made it a point to talk to people who weren’t talking and maybe felt alone in a drop-in setting. (P10)

Peers noted the value of social connections as a bridge to navigating the healthcare system.

I think social connections also extend to like healthcare connections you know? And getting different services you know? Because that does require you to leave your house and go out there. So, I think just the act of being a peer supporting someone to go to an appointment is enhancing a social connection. (P1)

Not only were social interactions helpful for clients to develop more ease in socializing, they represented very positive and rewarding experiences for clients. “Once they start having the experience of joy and finding people that they can talk to, it becomes so much easier to fix everything else.” (P10).

Part of why new social connections are so important is that many TAY clients are embedded in social contexts where those around them are also dealing with complex social determinants that impact their wellbeing.

I think humour. Because I think for clients, they’re always stuck in this, place where it’s like, its usually like bleak and things suck, you know? I think humour brings, or at least allows people to take a second away from all that mess and it causes people to open up more. And you know, you can joke around with people. And I think altogether, for me at least, I think it’s really important to get people out of their shell and have some fun… (P10)

Peers served as a stepping stone for clients to access larger spheres of social connections, with benefits beyond those of a clinical environment. Often, these interactions helped clients to overcome isolation or negative social environments and re-engage with society. “I think it’s really important that we get people out of the house and I think peers would be the perfect avenue for that specific task.” (P3)

I really believe that [the peer] role in that relationship is the bridging role, right. So it's like, if I'm up here, and I'm working with a youth who maybe isn't quite ready for therapy or case management, but they just kind of want to talk to someone that gets it. I think that's where the peer is the connecting link… (P2)

The value of peer-led programming is that young people are more connected to what is important and interesting to other young people.

We’ve started the DND group, dungeons and dragons. People who met at the drop in, who, a lot of them are on the spectrum, so they have a lot of issues socializing, but when DND comes into it, then it makes it easy for them to talk to people. (P10)

### CMOC 3

The third program theory describes a process whereby clients recognize peer advice as having significant value since it is based on lived experience. Further, peer recovery represents tangible proof that this advice can be effective and supports the development of client hope and increases the likelihood that they will apply recommendations. Finally, these perceptions motivate clients to apply strategies and experience success that supports increased self-efficacy. This theory is aligned with the Social Cognitive Theory.

A key piece of this process involves peers sharing experiential learning, or offering insight that they have gained through their experiences of coping with relevant issues. This communicates important information in very concrete terms and strengthens the validity of peer advice. “I find that people connect to stories more than they connect to facts or figures kind of naturally. To be able to kind of cushion an idea in a story, it communicates way better.” (P3)

I was able to tell him that I’ve experienced addiction, I’ve experienced issues at home, abuse and all these sorts of things and I really experienced a lot of situations, so giving him a little piece of that, I think it just is a reiteration of, ‘it is possible, and we can recover’ and there’s people who have taken steps to do that and there’s different avenues… I think it’s just giving them those options and telling them that “I’ve experienced it too” and I think that really gives them the hope and even just the seed of the idea. (P10)

Peers also discuss the critical insight that clients gain through the recognition of the utility and potential of successful solutions that peers have applied in their recovery journey. Clients see that the same path is a possible opportunity for them and this can change their perspective on whether there is value in attempting these strategies. “If I were to say like ‘don’t do this, because this will happen.’ You know, [clients] are never going to learn like that. They just don’t.” (P10).

Aspirational role modelling is a concept that describes this process whereby peers model positive coping behaviours and strategies that clients can then adopt. “Not only do I understand where you’re coming from, I am a potential future for you.” (P3) “Peers, by virtue of existing, show clients that this is another pathway for you.” (P1) Peers also describe how this awareness motivates clients to attempt the strategies offered, and this often leads them to experience success and to initiate a positive momentum forward.

Being able to share these lived experiences and kind of mentioning that there is a path behind me as well I find people tend to open up more because there is more understanding and validity to the interactions. And when you're able to validate the person using your own experiences and what they're doing seems effective I find really reinforces those skills that people are practicing. (P11)

Sharing these experiences can be a pivotal starting point for clients who are experiencing really intense challenges and who are having a hard time finding a way forward.

I think, just listening and knowing that the worker’s coming from a vulnerable place, and they're willing to meet you halfway. Just diving into the depths of the struggles. And it's, like, a critical starting point for the intervention. Especially when the distress is so high that the person just, they can't even fathom, how to move forward. And that can really, just help them navigate themselves through that. And, feel like their support is really joint. And they can just use the inspiration from what they've heard to find that confidence start to develop. (P8)

### CMOC 4

The final program theory describes the holistic features of peer support and how these can be used to enhance client autonomy and self-determination. As mentioned earlier, the TAY program was designed to support young people who are experiencing significant challenges and the peer service approach takes account of these social determinants along with individual client characteristics. Some clients were experiencing challenges because of developmental diagnoses and peers are able to create opportunities for these clients to engage. Challenges can be related to social norms, cultural beliefs, lack of opportunities and economic issues. These conditions can lead to perpetual invalidation that weakens client self-determination.

You get trapped in it if you don’t have the next step. Like, there’s so many people that I’ve known who have quit and kind of get their life together, but then they won’t find employment, and then what do you do now, well my life goes to drugs. You need that in place or you’re just going to fall back in. (P10)

There’s of course the newcomer mental health effect where there’s like, it’s doesn’t exist. There’s no words for it so it doesn’t exist socially. It doesn’t exist on the ground. And all of a sudden you’re here and you’re having these experiences of trauma and you can’t name them. (P1)

The TAY program is connected to other organizations and services to address client needs more holistically and directly. This ecological approach is translated through integrating peers as complementary to other services and to support increased access and engagement for clients that benefit from support that is easier to relate to: “Not only is it integrated so much within the program, but there’s so many connections within different agencies.” (P8)

So a peer can't save the world. A case manager can't save the world. A police officer can't save the entire country. But if we come together, and we put our heads together, and we figure out a way that you're going to do this, I'll do this, because that's what I'm good at. (P2)

Peers help to tailor services so that clients are offered what they need in the moment and processes are set up so that services are matched to client needs.

So it is getting to appointments or accessing services and peers can be involved to help them. Like we’ve had peers do exposure therapy around going out in public or being in public places. So it’s like a very skillful thing to do, but it’s outside of the time that a case manager has in order to do that. (P6)

In the TAY program, processes have been developed to support a warm transfer or enhance the circle of care surrounding clients. Previous to having these processes, there were many challenges around role clarity with respect to what the peer can offer the client. Often peers were placed with clients and there was little to no information offered to clients with respect to the purpose of the connection.

We developed a peer referral form so that the staff, when they’re considering engaging a peer would kind of give a thoughtful consideration to that. Like what’s the goal here? Like what’s the purpose of engaging a peer? And it would be around what skills does this peer have or what’s the specific thing they want to work on? And then they would bring that to me…but also they could express like I’d really like it if it could be [participant 4], or for it to be [participant 9]. Like I just think they would be a good match. Then we would talk to the peer to see if that really works for them. (P6)

We’ve worked with people before where we both attend the meeting and [participant 4] is able to interject with DBT skills that might be helpful for that person in the moment or applying them to a specific situation. So like some of it, if I’m meeting with a person individually and I see that they could benefit then it’s something I suggest. It’s also up to the client whether or not like they want to work with another person. (P12)

Adaptations that are achieved to address client services more holistically are informed by client preferences and offer clients opportunities to take control over goal development and their direction in recovery. This helps to empower clients and to develop their agency to move forward independently:

You get to choose and you get to take power and own what you want in your life and what you don't. And it's just about empowering those people. When they make those decisions, like wow, you know, like ‘You did that. Like, you just made that choice!’ (P2)

### Over-arching outcome: recovery

Both peers and clients describe recovery as both an individual and dynamic process. The path to recovery will look very different for each person, but generally has an aspect of incrementally building wellness, stability and independence. One of the goals of peer support is helping clients along their individual path of recovery.

I noticed over time working with people that there is a sort of like level of hope and … they are capable of developing their wellness and, like developing their stability. (P7)

I've also been in situations where I felt like a kind of hopelessness, and they can sort of see somebody who is doing these things, you know, like finding stability and finding wellness. And I think that they can see people start to go into a more like a wellness and recovery-based mind where they feel like that future is possible. (P1)

It can also help create, like, the goal, and like the things that they're really working on in their own recovery become a little bit more tangible…I think that's probably the major impact on that, where they can actually see concrete proof of different paths, or interventions or strategies that can be used and taking care of oneself or finding recovery in their wellness. (P5)

### Survey results

There were 187 clients who participated in TAY peer groups during the period of survey collection. Of these, 77 (41.2%) participated in the online survey. As seen in Table 3, survey respondents reflected a diverse sample that was largely representative of the client population within the TAY program, in that they were relatively young, primarily female, and while half identified as white, the remainder represented numerous ethnoracial identities. There was a range in length of time participating in the LOFT program, but the largest proportion of respondents were relatively new (0–6 months), and had been receiving peer services for 3–6 months (Table 3).

**Table 3:** Characteristics of survey participants, service use and client perceptions

*Participant characteristics*^a^	*n*	*%*	*M*	*SD*
Age			22.43	3.15
Gender				
Girl/woman	44	57		
Boy/man	14	18		
Alternative gender	18	23		
Racial background				
Asian	16	21		
Black	7	9		
Middle Eastern	4	5		
Other	5	6		
White	39	50		
*Service usage*				
Length of time as client at LOFT				
0–6 months	32	42.6		
6–12 months	9	12.0		
1–2 years	18	24.0		
over 2 years	16	21.3		
Length of time receiving peer services				
0–3 months	32	43.2		
3–6 months	8	10.8		
6–12 months	12	16.2		
Over 1 year	22	29.7		

^a^There was very little missing data for demographic information. Only one respondent preferred not to provide gender. Two indicated that they did not know their ethnoracial background, three preferred not to answer and one respondent skipped this question. Percentages are based on valid data

Descriptive statistics regarding perceptions of peer supports generally supported the tenets of the program theories derived. Specifically, as seen in Table 4, the majority of client respondents reported that they related well and felt comfortable with their peer supports. In addition, when asked about their level of agreement with the statement: “I see my peer supporter as a positive role model”, most clients perceived their peer as a positive role model. For youth efficacy and empowerment, average scores were slightly lower than the norm. Average self-rated mental health and life satisfaction were both very low in contrast with typical scores in the Canadian population [55]. We were unable to source norms for the therapeutic alliance scale, however, ratings appeared to be quite high with an average of 3.22 out of a possible 4.

**Table 4:** Correlations Between Context, Mechanism and Outcome Variables

Variable	M (SD)	1	2	3	4	5	6
1. Relating to peers	3.07 (0.81)						
2. Comfort with peers	3.37 (0.67)	.51**					
3. Peers as role models	4.20 (0.75)	.47**	.59**				
4. Therapeutic alliance	3.22 (0.57)	.40**	.59**	.54**			
5. Self-efficacy	2.49 (0.63)	.14	.29*	.06	.13		
6. Self-rated mental health	2.14 (0.93)	.08	.03	−.08	−.10	.40**	
7. Self-rated life satisfaction	2.82 (1.00)	−.00	−.02	−.02	−.06	.40**	.67**

**p < .01 (2-tailed)

* *p* < .05 (2-tailed)

As seen in Table 4, Pearson correlations indicate that self-reported client comfort with peer supports was positively associated with youths’ feelings of self-efficacy and empowerment (r = .29 *p* <. 05). Such reports of self-efficacy/empowerment were further correlated with both life satisfaction and mental health. Thus, although the variables representing the context/mechanism processes were not directly related to mental health outcomes, consistent with CMOC3, they might elicit improved outcomes through client empowerment. Therapeutic alliance was positively correlated with client perceptions of peers as role models, peer relatability, and client comfort-level with peers. Finally, there was a positive correlation between length of time as a client of TAY and self-rated mental health (r = .23 *p* < .05), but not length of time receiving peer services.

21 (27.7%) of respondents said that the peers helped them to develop new social connections and several respondents described these social connections. The following are some of their responses

“I have had the opportunity to meet new people, because of the peer support program.” “This has helped me learn new skills and experiences. This has been beneficial to me.”

“I have created genuine friendships where my peers do not judge me for my struggles that I go through and not to have the worry of somebody judging me for having anxiety or depression is very supportive.”

“Being a part of this group has given me a sense of support and community.”

“It helps to know other people feel the way I do and that you can have a mental illness and still get somewhere in your life.”

“I am pretty reclusive so even just talking to the peer support worker is progress, and its also nice to be able to reflect with someone on how I experience social interaction in the world.”

## Discussion

This study was designed to examine how and why the TAY peer services are effective in order to better understand how to design effective peer services. This study has begun to identify the interconnections that function to create client impacts within youth peer practice and these findings can contribute to supporting the development of more effective programming in the future. This study identified four program theories within the LOFT-TAY program that were related to client outcomes, along with an over-arching context that was foundational to each theory and one broad outcome that served as the long-term objective for each of the four processes.

This study involved a participatory-realist evaluation of a specific program context, therefore the results should be examined in other settings to identify whether they are valid and applicable to youth peer support more generally. Our findings support the development of peer programs that place a focus on stigma reduction (CMOC1), the expansion of social connections (CMOC2), the promotion and practice of practical coping strategies (CMOC3) and the development of client autonomy (CMOC4). They also suggest that programs should be tailored to client needs and address key social determinants (CMOC4). Programs should provide opportunities to practice social skills (CMOC2) and highlight that peers serve as esteemed examples (CMOC1) of potential recovery (CMOC3). Programs should be built on the foundation that peer lived experience can create a safe space for clients and leverage this lived experience to enhance engagement and ultimately, recovery. These elements can be explicitly integrated into training manuals to enhance peer role clarity and development of organizational supports to create these conditions (see Halsall et al.: “You can create a little bit more closure in your own story when someone really connects with it”: Exploring how involvement in youth peer support work can promote peer development, submitted).

Although the body of literature on youth peer support in mental health services is relatively small, there is considerable support for the theories that emerged in our data. For example, in line with the over-arching context and outcome, previous studies have identified the need for the development of safe space within youth peer support programs [24, 56, 57] and their effectiveness in moving clients toward recovery [1, 24].

Moreover, each of the emergent theories align with broader theoretical frameworks that represent general understandings of social processes (Social Cognitive Theory, Social Identity Theory, the Bioecological Model and Self-determination Theory). As described in the methods, the Social Cognitive Theory and the Social Identity Theory were identified at the outset as meriting greater exploration as they are implicated in the peer support and mental health stigma literature. As program theories were refined, the Self-Determination Theory and the Bioecological Model were identified as also having relevance for explaining how and why the TAY program is effective.

### Positive identity development through identification with peers (CMOC1)

CMOC1 maintains that clients develop a more positive identity through the recognition that they share a social reference group with peers. Consistent with our findings, in a randomized controlled trial of a mental health peer support service designed to reduce the stigma of mental illness, the intervention group experienced reduced self-stigma, reduced stigma-related stress and enhanced quality of life [22–24]. It is interesting to note that although the intervention enhanced participant capacity to cope with stigma stress, it did not impact their perception of stigma as a stressor, which is likely a reflection of stigma as a societal norm in general and that this is potentially not feasible to change through self-disclosure skills training [22]. By extension, it may be unrealistic to anticipate that awareness-raising stigma reduction campaigns that follow an educational or skills-based approach such as this would be successful at a population level. Investments should be focused on interventions, such as youth peer support, that target those most affected and support them in overcoming stigma-related stress.

CMOC1 reflected concepts derived from Social Identity Theory [32], and was refined through two rounds of interviews. This program theory conceptualizes that self-stigma can be diminished through the recognition that peers are a member of a shared reference group. This group is held in higher esteem as peers are positive representative members and through this process, clients develop a more positive sense of identity. We describe related findings in Halsall and colleagues [“You can create a little bit more closure in your own story when someone really connects with it”: Exploring how involvement in youth peer support work can promote peer development, submitted], whereby, peers overcome stigma through their roles as peer supporters. Peers recognize that their lived experience of a mental health issue or other challenge is the pivotal attribute that serves to support their insight in contributing to other wellness and plays a key role in their success as a peer. Therefore, this characteristic that initially served to stigmatize them has now become valuable, both to them and others.

### Enhanced social connections (CMOC2)

CMOC2 highlights the function within peer practice that supports the development of client social connections. In support of CMOC2, there is evidence to suggest that that peer support approximates friendship [58]. Other youth peer support programs have demonstrated increased client social functioning and personal relationships [1], and decreased social withdrawal [24]. Participants from a peer support intervention for young people experiencing homelessness have highlighted the importance of relational supports and the development of social connections [25] particularly for young people who are transitioning away from homelessness as this often leaves them in a state of social isolation.

CMOC2 provides support for the bioecological model [45]. The bioecological model is a theoretical frame that conceptualizes development as an interactional process between the individual and multiple systems of influence. The theory highlights a reciprocal interaction between the individual and the environment resulting in mutual influence. Enhancing social connections through TAY peer services signifies that the services are having an impact on a significant influential developmental context, primarily, the peer context. Through the practice of social skills and reintroduction to healthy social relationships, the program is supporting the development of a positive social context that may have much broader ramifications on wellbeing and resilience in moving toward recovery. This finding may be more specific to TAY clients who are coming from homelessness, social isolation or other challenging contexts that are lacking in informal social supports. Similar findings were derived within other research on peer support in youth homelessness populations (see [2, 57]).

### Observational learning (CMOC3)

The third program theory references the development an interaction wherein clients recognize the significance of peer recommendations and experience increased self-efficacy through putting these recommendations into practice. This is aligned with research that has highlighted that within youth peer services, participants felt the stories regarding peer lived experience were valuable and perceived peers as positive role models [24]. Youth peer support programming has been found to support the development of coping skills [25, 57] a reduction in hopelessness, and increased optimism [24].

The social cognitive theory was identified as being relevant to peer support practice earlier in the design of this study. Both our qualitative and quantitative findings pertaining to CMOC3 support this theory. Of particular relevance is the process of social modelling whereby clients “pattern their styles of thinking and behaving after the functional ones exemplified by others” ( [26] , p.11). In addition, the development of self-efficacy through the application of recommended strategies is another element of the program theory that represents an earlier outcome. This process supports continued engagement in strategies that move clients further forward in their recovery. We found that higher client self-efficacy, as measured by the YES-MH, was associated with higher self-rated mental health and overall life satisfaction.

Our quantitative analyses identified positive associations between client comfort with peer supports and self-efficacy, which in turn was associated with greater mental health. We did not find associations between the other processes that emerged as important in the qualitative analyses. This may have resulted because we used a cross-sectional design assessing outcomes at a single time point. It is possible (indeed likely) that impacts would be more evident over time spent in services. In addition, TAY clients were often dealing with significant hardships and might continue to experience lower mental health and life satisfaction, despite benefitting from TAY peer services. We did find a positive association between the length of time receiving services and self-rated mental health, however, the majority of clients were new, therefore, this may have attenuated associations between program theory context, mechanism and outcome variables.

### Enhanced autonomy and empowerment (CMOC4)

Finally, CMOC4 takes account of the holistic and adaptable aspects of peer practice and the mechanisms through which these can be applied to enhance client autonomy and self-determination. Likewise, other youth peer interventions have been found to enhance participant empowerment [1] and autonomy [59]. Kidd and colleagues [2] identified that participants who were more highly engaged with peers were more likely to demonstrate autonomy through becoming involved in employment, education, and/or volunteering. Participants noted that the program built their confidence as they were able to take on responsibility in slow incremental steps. CMOC4 conceptualizes clients in a holistic perspective that acknowledges a range of social determinants that affect them and highlights the adaptation that is made within services to meet client needs in a more comprehensive manner. These aspects of peer practice are consistent with the bioecological model as this theory places an emphasis on the strength of contextual influences as well as the relevance of individual agency. Each client brings a range of complex challenges that they are coping with in their environment and these considerations are important for making program adaptations that are effective. Further, a recognition of these issues and opportunity to identify future directions are important for supporting client self-determination [40].

Finally, several program theories lend support to the Self-determination Theory [60] with respect to the satisfaction of the three basic needs: mastery (CMOC3), autonomy (CMOC4) and relatedness (CMOC2).

### Future directions

Following realist methodology, these program theories should be tested through a more thorough account of client perspectives on outcomes [61] as well as a more thorough measurement of outcome patterns through quantitative data [17]. If these findings prove to be replicable, then the program theories will be useful to serve as the foundation for the identification of critical components within youth peer support, new program design and training development. Each of these theories places a significant emphasis on social influences, and this is likely a reflection of the significant strength of peer services over other modicums of practice, and their relevance to social contexts outside of structured programming. Through the identification of explanatory mechanisms, this research offers a clear understanding of the underlying theory of change that underpins effective youth peer support and can be used to strengthen implementation and effectiveness across programs. Continued involvement from both peers and clients will be critical to refining and improving our understanding of youth peer practice and will enhance outcomes across services.

Our findings are relevant for research examining interventions to reduce stigma. There is a significant amount of investment that has been targeted at mental health stigma campaigns, yet there has been a negligible influence on societal perceptions of mental health. In terms of mental illness stigma reduction that is targeted toward young people, the evidence base is lacking and many researchers do not provide full support for existing interventions [38]. Our findings are of particular significance regarding the perceptions of young people experiencing mental health and substance use issues. Through the development of positive relationships with a peer group experiencing similar challenges, TAY clients were able to experience relief from the significant hardships that are related with stigma. Despite there being no significant change to societal perceptions, they felt that their self-perceptions of stigma improved nonetheless. As discussed previously, peer practice has been found to reduce client self-stigma as well as stigma-related stress [24]. Further, researchers have recommended that investigations focus on identity-related concepts to reduce stigma-related barriers for young people [38]. In the future, investments in initiatives to improve stigma should focus on peer-related approaches that target groups who are stigmatized, rather than raising awareness with the general population.

### Strengths and limitations

This study was initiated a few months before the COVID-19 pandemic, whereby many services and engagement practices transitioned to a virtual model. Subsequently, peer roles, programming and data collection methods were shifted to an online approach. This transition made it more difficult to recruit clients for the survey, therefore we were not able to examine outcome patterns or collect information about non-respondents for comparison.

This study benefitted from significant strengths. The TAY program was designed using a participatory and strengths-based lens [62], therefore, participatory evaluation was a natural complement for this program philosophy. Combining the realist and participatory methods helped with organizational buy-in to the realist approach. The combination of the two methods supported the blending of insight from lived experience and theory. Our participatory approach was comprehensive (see [3]), wherein all TAY program staff were involved in key decisions and reviewed and provided feedback on the findings. Daley was a co-researcher and was involved in design, data collection, staff engagement, analysis, interpretation, development of knowledge mobilization products, writing and facilitation of presentations. Daley made key contributions that were critical in the study development, selection of tools and development of codes and final program theories. Her insight was important for adapting findings to peer practice terminology and to ensure that measures would be acceptable to respondents and appropriate for the TAY program context. Daley was able to enhance communication with LOFT and as a result, our partnership was more influential and effective.

## Conclusion

This study applied participatory and realist methods to an in-depth examination of a youth peer support program for young people coping with complex mental health and substance use challenges. It represents an exemplar of the integration of two complementary methods and can be used to support design of future evaluation studies that blend insight from lived experience, practice and theory. This research contributes to an expanding body on youth peer support in mental health and connects peer practice with several social theories. In particular, this research enhances understanding regarding how and why peer support might be beneficial and lays the foundation to begin to more formally test these theories and utilize them in future program design. Finally, our findings can inform future interventions to support young people coping with the stigma of mental illness and other related challenges.

## Data Availability

The data that support the findings of this study are available on request from the corresponding author. The data are not publicly available due to privacy or ethical restrictions.
